# Histopathological evaluation of local effects of radioactive iodine seeds in axillary lymph nodes in clinically node-positive breast cancer treated with neoadjuvant systemic therapy

**DOI:** 10.1016/j.breast.2026.104746

**Published:** 2026-03-04

**Authors:** Florien J.G. van Amstel, Janine M. Simons, Loes Kooreman, Melissa Lenaerts, Sander M.J. van Kuijk, Lars H.P. Murrer, Cornelis M. de Mooij, Ernest J.T. Luiten, Carmen C. van der Pol, Linetta B. Koppert, Marjolein L. Smidt, Paul J. van Diest, Thiemo J.A. van Nijnatten, REFINE Study Group

**Affiliations:** a Department of Radiology and Nuclear Medicine, Maastricht University Medical Centre+, Maastricht, the Netherlands; b GROW – Research Institute for Oncology and Reproduction, Maastricht University, Maastricht, the Netherlands; c Department of Radiotherapy, Erasmus MC Cancer Institute, University Medical Centre Rotterdam, Rotterdam, the Netherlands; d Department of Pathology, Maastricht University Medical Centre+, Maastricht, the Netherlands; e Department of Surgery, Maastricht University Medical Centre+, Maastricht, the Netherlands; f Department of Clinical Epidemiology and Medical Technology Assessment, Maastricht University Medical Centre+, Maastricht, the Netherlands; g Department of Radiation Oncology (Maastro), GROW Research Institute for Oncology and Reproduction, Maastricht University Medical Centre+, Maastricht, the Netherlands; h Department of Surgery, Haaglanden Medical Centre, The Hague, the Netherlands; i Department of Surgery, Amphia Hospital Breda, Breda, the Netherlands; j Department of Surgery, College of Medicine and Health Sciences, United Arab Emirates University, Al Ain, Abu Dhabi Emirate, United Arab Emirates; k Department of Surgical Oncology, Alrijne Hospital, Leiderdorp, the Netherlands; l Department of Surgical Oncology, University Medical Centre Rotterdam, Rotterdam, the Netherlands; m Department of Pathology, University Medical Centre Utrecht, Utrecht, the Netherlands; n Dutch Expert Centre for Screening (LRCB), Nijmegen, the Netherlands

**Keywords:** Breast neoplasms, Lymph nodes, Neoadjuvant therapy

## Abstract

**Background:**

In clinically node-positive (cN+) patients, radioactive iodine (^125^I) seeds can be considered to mark metastatic axillary lymph nodes before neoadjuvant therapy (MARI-procedure) but potential therapeutic effects remain unclear. This study compared histopathologic features between MARI (containing ^125^I seed) and non-MARI nodes and evaluated the association between ^125^I seed absorbed radiation dose and histopathologic features in MARI nodes.

**Methods:**

This retrospective study included cN+ patients from the Radioactive Iodine Seed placement in the Axilla with Sentinel lymph node biopsy (SLNB) (RISAS)-trial (NCT02800317). Non-MARI nodes comprised SLNB or completion axillary lymph node dissection nodes. An expert breast pathologist reviewed MARI and non-MARI nodes with residual disease for the presence of fibrosis, mucin pools, foamy histiocytes, capsular invasion, and residual disease (size). Differences were analysed using multilevel logistic regression. The effect of ^125^I seed absorbed radiation dose (Gy) in MARI nodes was evaluated using univariable logistic regression. Results are presented as odds ratios (OR) with 95% confidence intervals (CI).

**Results:**

106 MARI and 326 non-MARI nodes were included from 127 patients. Fibrosis, foamy histiocytes, and macrometastases were significantly more frequent in MARI compared to non-MARI nodes, with ORs of 21.5 (95% CI: 9.51-57.12), 17.1 (95% CI: 3.53-82.44), and 2.3 (95% CI: 1.22-4.37). In MARI nodes, ^125^I seed absorbed radiation dose had no significant effect on histopathologic features, with ORs between 1.0 and 1.1.

**Conclusions:**

Fibrosis, foamy histiocytes, and macrometastases were significantly more present in MARI compared to non-MARI nodes. Moreover, ^125^I seed absorbed radiation dose had no significant effect on histopathologic features in MARI nodes.

## Highlights

• Analysis of local effects of ^125^I seed-marked nodes in node-positive breast cancer.
• Nodes with and without ^125^I seed with residual disease were compared.
• ^125^I seed-marked nodes showed more fibrosis, foamy histiocytes, and macrometastases.
• ^125^I seed absorbed radiation had no significant effect on histopathologic features.

## 1 Introduction

The use of neoadjuvant systemic therapy (NST) in clinically node-positive (cN+) breast cancer patients leads to approximately one third of the cN+ patients achieving an axillary pathologic complete response (pCR) [1,2]. Consequently, less invasive axillary surgical staging procedures were developed to identify axillary pCR after NST: the excision of the pre-NST marked metastatic lymph node, sentinel lymph node biopsy (SLNB), and approaches that combine these procedures such as targeted axillary dissection or radioactive iodine seed placement in the axilla with sentinel lymph node biopsy (RISAS) [[3], [4], [5], [6], [7]]. In case radioactive iodine (^125^I) seeds are used to mark metastatic lymph nodes pre-NST, this is called the MARI-procedure [8].

The ^125^I seed is a source of radiation that emits gamma rays and can therefore also be used to deliver targeted radiation. For instance, in prostate cancer, up to 100 ^125^I seeds are placed inside the prostate for curative treatment (brachytherapy) [9,10]. At present, in breast cancer, ^125^I seeds are mainly used for diagnostic purposes, specifically to mark primary tumours and metastatic lymph nodes, and can be placed either before or after the completion of NST. For this diagnostic purpose, ^125^I seeds with a lowered activity (0.3-11.1 megabecquerel (MBq)/0.01-0.3 millicurie (mCi)) are used that are expected to have no or minimal local effect but that (due to the relatively long half life time of 60 days) still have enough activity after completion of NST (approximately 6 months) to be detected by the gamma-probe [8].

However, the use of ^125^I seeds to mark primary tumours and metastatic lymph nodes pre-NST remains debated for two main reasons. First, the radioactivity of ^125^I seeds raises regulatory concerns. In The Netherlands, ^125^I seeds are legally permitted to remain in situ during the entire course of NST [11]. In contrast, in some countries, regulation prohibits leaving a ^125^I seed in place for more than a few days to weeks due to radiation concerns for diagnostic purposes (for instance, up to 5 days in The United States of America) [[11], [12], [13]]. Second, the effect of ^125^I seed radiation on tumour cells and local tissue remains unclear. Previous studies have been limited to in vitro models within brachytherapy settings and have primarily focused on tumour types other than breast cancer [[14], [15], [16], [17]].

Yet, the increasing use of NST in cN+ patients requires accurate pathologic evaluation of treatment response [18]. Pathologic response after surgery informs adjuvant treatment and prognosis and is routinely assessed using histopathologic features such as fibrosis, mucin pools, or foamy histiocytes [19,20]. However, as both NST and local radiation exposure can induce fibrosis and inflammatory changes, the ^125^I seeds might mimic NST-induced tumour regression [21,22]. To guide appropriate adjuvant treatment, it is important to know whether histopathologic features associated with tumour regression reflect the therapeutic effects of NST, rather than potential radiation effects of ^125^I seeds.

This study focused specifically on the local effects of ^125^I seeds in their use to mark metastatic lymph nodes pre-NST. First, this study investigated whether there is a difference in histopathologic features between lymph nodes containing the ^125^I seed (MARI nodes) compared to lymph nodes without a ^125^I seed (non-MARI nodes). Second, the association between ^125^I seed estimated absorbed radiation dose and histopathologic features in MARI nodes was evaluated.

## 2 Materials and methods

### 2.1 Study design and patients

This retrospective study used data from cN+ patients who participated in the prospective multicentre noninferiority RISAS trial (NCT02800317; METC number 2016-412). Patients participating in the RISAS trial were female patients aged ≥18 years with pathologically proven cN+ breast cancer (cT1-4 and cN1, cN2, or cN3b) and received NST prior to breast and axillary surgery. NST regimens generally consisted of anthracyclines and/or taxanes. For patients with HER2-positive breast cancer, additional targeted therapy (trastuzumab alone or trastuzumab and pertuzumab) was added. As part of the RISAS trial, axillary surgery consisted of the MARI-procedure combined with SLNB, followed by completion axillary lymph node dissection (cALND) within a single surgical procedure. Written informed consent had been obtained from all patients during the RISAS trial. A detailed description is provided in the RISAS study protocol [23] and in the publication of the primary results of the RISAS trial [3].

In this retrospective study, all resected lymph nodes from patients who had residual disease following NST were centrally reviewed, and only MARI and non-MARI nodes containing residual disease were included in this analysis. Pathology reports and lymph node samples were collected from all local institutes participating in the RISAS trial through PALGA, the nationwide network and registry of histopathology and cytopathology in The Netherlands [24]. The privacy and scientific committee of PALGA provided approval for this study (protocol number 2022-109).

### 2.2 The MARI-procedure

Patients underwent ultrasound-guided placement of a ^125^I seed in the pathologically proven metastatic lymph node at baseline prior to the start of NST. In case multiple suspicious lymph nodes were identified on baseline axillary ultrasound, the lymph node with the most suspicious morphology was marked with the ^125^I seed. After completion of NST, the lymph node containing the ^125^I seed was removed and confirmed with a gamma probe and/or specimen radiograph [3].

### 2.3 Absorbed radiation dose

To estimate the local absorbed radiation dose (Gy) around the ^125^I seed, a point source model was employed accounting for ^125^I seed radioactivity at time of placement (MBq), the duration the ^125^I seed remained in place (days), tissue attenuation, and a fixed reference distance of 5 mm from the ^125^I seed (Table S1, Fig. S1). The fixed reference distance of 5 mm was chosen because the observed histopathologic features were not located directly near the ^125^I seed cavity. Using the 5 mm distance allows the estimated absorbed radiation dose to better represent the tissue region where the histopathologic effects were assessed, enabling a more accurate correlation between the estimated absorbed radiation dose and observed histopathologic changes. However, important to note is that the 5 mm is a conservative estimate, as the ^125^I seed dose increases steeply at smaller distances from the ^125^I seed. The absorbed radiation dose was calculated in a subset of patients for whom data on ^125^I seed radioactivity at time of placement and duration the ^125^I seed remained in place were complete.

### 2.4 Histopathologic evaluation

**Fig. 1:**
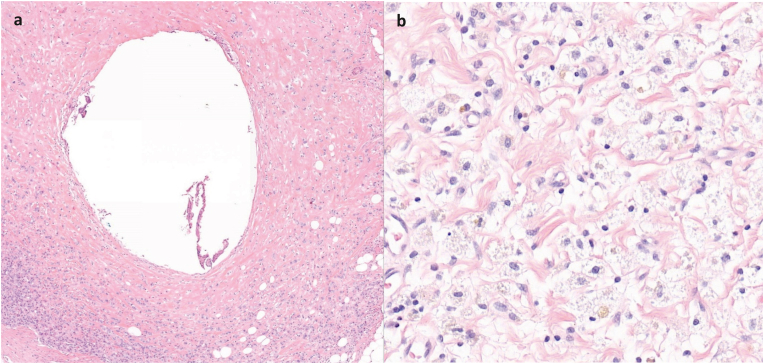
Digital haematoxylin and eosin-stained tissue sections from a MARI node: (a) ^125^I seed cavity containing giant cells surrounded by scattered tumour cells within fibrotic tissue; (b) foamy histiocytes.

The haematoxylin and eosin samples of all resected lymph nodes from patients who had residual disease were retrieved from all local institutes that participated in the RISAS trial and scanned using the 3DHistech Panoramic p1000 scanner. Using 3DHistech slide viewer, all digital samples were centrally reviewed by a single expert dedicated breast pathologist (P.J.v.D) with >30 years of experience in breast pathology. In this central review, lymph nodes removed at SLNB and cALND (that did not contain the ^125^I seed) were considered non-MARI nodes. The lymph node from which the ^125^I seed was removed by the local pathologist was considered a MARI node. If the sentinel lymph node was the same as the MARI lymph node (and contained the ^125^I seed), this node was considered a MARI node. To determine local response to the ^125^I seed, histopathologic features including the presence of fibrosis (i.e. yes/no), mucin pools (i.e. yes/no), foamy histiocytes (i.e. yes/no), capsular invasion (i.e. yes/no), and size of residual disease (i.e. isolated tumour cells (ITC)/micrometastases/macrometastases) were assessed in both MARI and non-MARI nodes. The MARI nodes were additionally assessed on the presence of a cavity caused by the ^125^I seed and if present, the distance (mm) between the ^125^I seed cavity and the nearest tumour deposit was documented. Because additional cavities were occasionally observed, likely due to artifacts from histopathologic preparation, a ^125^I seed cavity was only considered as such if giant cells were present within the cavity, which is indicative of a foreign-body reaction (Fig. 1).

### 2.5 Statistical analysis

To explore differences in histopathologic features between MARI and non-MARI nodes, univariable multilevel logistic regression analyses were performed to account for data clustering (MARI vs. non-MARI nodes nested within patients). Five models were constructed, each with type of lymph node (MARI vs. non-MARI) as the independent variable and a different dependent variable: the presence of (1) fibrosis, (2) mucin pools, (3) foamy histiocytes, (4) capsular invasion, and (5) size of residual disease. The variable size of residual disease was dichotomised into a binary outcome (ITC/micrometastases vs. macrometastases).

To explore whether ^125^I seed estimated absorbed radiation dose affected histopathologic features in MARI nodes, only MARI nodes were included in the analysis. Five univariable logistic regression models were constructed, each with ^125^I seed estimated absorbed radiation dose as the independent variable. The five models used the same dependent variables as those used in the multilevel analyses.

Missing data were included in the analyses coded as ‘unknown’. Results of the logistic regression models were expressed as odds ratios (OR) with 95% confidence intervals (CI). Logistic regression analyses were performed only for variables with sufficient events (>10) to ensure model stability. All statistical tests were two-sided, and P < 0.05 established the level of significance. Statistical analyses were performed with R Statistical Software (v4.4.2; R Core Team (2025). R Foundation for Statistical Computing, Vienna, Austria).

## 3 Results

### 3.1 Baseline characteristics

This study included 127 patients with axillary residual disease following NST, comprising a total of 2043 identified lymph nodes, of which 118 MARI and 1925 non-MARI nodes. Only lymph nodes with residual disease were included in the final analyses: 106 (89.8%) MARI and 326 (16.9%) non-MARI nodes. The median ^125^I seed radioactivity at the time of placement was 6.1 MBq (0.17 mCi, range: 1.22-10 MBq) and the ^125^I seeds were in place for a median of 168 days (range: 71-238 days). The mean ^125^I seed estimated absorbed radiation dose at 5 mm was 12.4 Gy. In MARI nodes in which a ^125^I seed cavity was identified (77/106, 72.6%), the mean distance between the ^125^I seed cavity and the nearest tumour deposit was 0.9 mm. Baseline characteristics of the total study population are listed in Table 1.

**Table 1:** Baseline characteristics.

Variables	Overall (n = 127)
**Age (years)**	
Median [range]	51 [28-75]
**^125^I seed radioactivity (MBq)**^a^	
Median [range]	6.1 [1.22-10]
**^125^I seed placement (days)**	
Median [range]	168 [71−238]
**Clinical T-status**	
cTx	1 (0.8)
cT1	14 (11)
cT2	78 (61.4)
cT3	32 (25.2)
cT4	2 (1.6)
**Clinical N-status**	
cN1	95 (74.8)
cN2	26 (20.5)
cN3b	6 (4.7)
**Oestrogen receptor**	
Negative	28 (22)
Positive	99 (78)
**Progesterone receptor**	
Negative	47 (37)
Positive	80 (63)
**HER2 receptor**	
Negative	104 (81.9)
Positive	23 (18.1)
**Breast cancer subtype**	
HR+/HER2-	81 (63.8)
HR+/HER2+	18 (14.2)
HR-/HER2+	5 (3.9)
Triple negative	23 (18.1)

Values in parentheses are percentages unless indicated otherwise. ^125^I seed, radioactive iodine seed; HR, hormone receptor; HER2, human epidermal growth factor receptor 2.

a At time of placement in 87 patients with 87 MARI nodes.

### 3.2 Histopathologic difference between MARI and non-MARI nodes

Table 2 presents a descriptive overview of histopathologic differences between MARI and non-MARI nodes, and Table 3 shows results of the regression models used to examine the histopathologic differences between MARI and non-MARI nodes. Multilevel regression could not be performed for the variable mucin pools due to insufficient number of events. Significantly increased ORs of 21.5 (95% CI: 9.51-57.12), 17.1 (95% CI: 3.53-82.44), and 2.3 (95% CI:1.22-4.37) were observed for fibrosis, foamy histiocytes, and macrometastases in MARI compared to non-MARI nodes (Table 3).

**Table 2:** Overview of the differences in histopathologic features between MARI and non-MARI lymph nodes with residual disease.

Histopathologic features	MARI nodes (n = 106)		*Radioactivity* *MARI nodes (n = 87)*	Non-MARI nodes (n = 326)	
			*Estimated absorbed radiation dose (Gy)*^a^		
**Fibrosis**					
Present	84	(79.2)	*12.4 ± 3.7*	118	(36.2)
Absent	22	(20.8)	*12.1 ± 5.2*	208	(63.8)
**Mucin pools**					
Present	1	(0.9)	*n/a*	2	(0.6)
Absent	105	(99.1)	*12.4 ± 4.0*	324	(99.4)
**Foamy histiocytes**					
Present	16	(15.1)	*12.2 ± 3.6*	11	(3.4)
Absent	90	(84.9)	*12.4 ± 4.2*	315	(96.6)
**Capsular invasion**					
Present	26	(24.5)	*13.8 ± 3.5*	62	(19)
Absent	80	(75.5)	*12.0 ± 4.1*	264	(81)
**Residual disease**					
ITC	3	(2.8)	*13.2 ± 0.1*	20	(6.1)
Micrometastases	29	(27.4)	*12.5 ± 4.2*	109	(33.5)
Macrometastases	74	(69.8)	*12.3 ± 4.1*	197	(60.4)

Values in parentheses are percentages. MARI, marking axillary lymph node with radioactive iodine seed; ITC, isolated tumour cells.

a ^125^I seed estimated absorbed radiation dose (Gy) expressed as mean ± standard deviation.

**Table 3:** Multilevel univariable logistic regression evaluating the differences in histopathologic features between MARI and non-MARI nodes with residual disease.

Multilevel univariable regression		OR	95% CI	*P* value
*Model 1*	**Fibrosis**			
	non-MARI lymph nodes	1 [ref]		
	MARI lymph nodes	21.5	9.51-57.12	**<.001**
*Model 2*	**Foamy histiocytes**			
	non-MARI lymph nodes	1 [ref]		
	MARI lymph nodes	17.1	3.53-82.44	**<.001**
*Model 3*	**Capsular invasion**			
	non-MARI lymph nodes	1 [ref]		
	MARI lymph nodes	1.5	0.81-2.96	0.191
*Model 4*	**Residual disease**^a^			
	non-MARI lymph nodes	1 [ref]		
	MARI lymph nodes	2.3	1.22-4.37	**.012**

OR, odds ratio; CI, confidence interval; MARI, marking axillary lymph node with radioactive iodine seed; REF, reference.

a Residual disease was categorised as a binary outcome: ITC/micrometastases vs. macrometastases.

### 3.3 Effect of ^125^I seed estimated absorbed radiation dose on histopathologic features in MARI nodes

Table 4 shows results of the regression models that were performed to assess the effect of ^125^I seed estimated absorbed radiation dose on histopathologic features in MARI nodes. For all outcomes, the ORs associated with ^125^I seed estimated absorbed radiation dose were close to 1 (range: 1.0-1.1), without statistically significant outcomes (Table 4).

**Table 4:** Univariable logistic regression evaluating the association between^125^I seed radioactivity and histopathologic features in MARI nodes.

Univariable regression		OR	95% CI	*P* value
*Model 1*	**Fibrosis**			
	^125^I seed absorbed dose^a^	1.0	0.90-1.16	0.745
*Model 2*	**Foamy histiocytes**			
	^125^I seed absorbed dose^a^	1.0	0.86-1.14	0.895
*Model 3*	**Capsular invasion**			
	^125^I seed absorbed dose^a^	1.1	0.99-1.30	0.083
*Model 4*	**Residual disease**^b^			
	^125^I seed absorbed dose^a^	1.0	0.88-1.11	0.817

OR, odds ratio; CI, confidence interval; ^125^I seed, radioactive iodine seed.

a ^125^I seed estimated absorbed radiation dose in Gy.

b Residual disease was categorised as a binary outcome: ITC/micrometastases vs. macrometastases.

## 4 Discussion

This unique retrospective study demonstrated that fibrosis, foamy histiocytes, and macrometastases were significantly more present in MARI compared to non-MARI nodes and showed that in MARI nodes, ^125^I seed estimated absorbed radiation dose had no significant effect on histopathologic features.

With the increasing use of NST in cN+ patients, accurate pathologic evaluation has become more important as response to NST guides adjuvant treatment and determines prognosis [18]. Generally, tumour regression features associated with therapeutic effects of NST include fibrosis, foamy histiocytes, mucin pools, or giant cells [19,20]. However, these features may also occur as a response to biopsy or clip placement and can be misinterpreted as therapeutic effects of NST [25]. Clark et al. described histopathologic features associated with placement of hydromark clips prior to NST, placement of ^125^I seeds after NST, and therapeutic effects of NST. The site of a hydromark clip was marked by fibrosis and histiocyte accumulation, the site of the ^125^I seed showed acute hemorrhage and inflammation, and they described NST-induced changes as loose fibroelastosis, dense fibrosis, and macrophage aggregates since these features were more diffuse (i.e. present in multiple areas within the lymph node and not only near the clips) [26]. Similarly, Bossuyt et al. stated that granuloma formation may occur around radiologic clips within lymph nodes [25]. The present study found that MARI nodes exhibited significantly more fibrosis and foamy histiocytes than non-MARI nodes. This may suggest a mechanical reaction to the ^125^I seed, as non-MARI nodes with residual disease did not show similar changes.

If the (lowered) radioactivity of ^125^I seeds used to mark metastatic lymph nodes pre-NST would have a therapeutic effect, one might expect improved local tumour response rates in affected lymph nodes that carried the ^125^I seeds. However, in this study, macrometastases were significantly more present in MARI nodes compared to non-MARI nodes following NST. This finding is largely explained by selection bias: nodes selected for the MARI-procedure appeared as the most suspicious lymph node on baseline axillary ultrasound and therefore typically have higher tumour burden (i.e. macrometastases). Importantly, when analysing the effect of ^125^I seed estimated absorbed radiation dose, no effect was found with histopathologic features in MARI nodes, including size of residual disease. From a radiobiological perspective, the absence of a dose-response relation is plausible. The ^125^I seeds used to mark metastatic lymph nodes pre-NST typically have a lowered activity of 0.3-11.1 MBq [11,12,27]. For comparison, therapeutic prostate brachytherapy achieves a cumulative tumour dose of 100-140 Gy using multiple seeds (up to 100), each with an activity of approximately 11-22 MBq [10,[28], [29], [30]]. Moreover, the absorbed radiation dose of ^125^I seeds decreases rapidly with distance. A review of the revised U.S. NRC licensing guidance reported that the absorbed radiation dose falls below 2 Gy only at more than 1 cm from the ^125^I seed surface, remaining below the thresholds generally associated with acute cytotoxic effects in standard radiotherapy [31]. Interestingly, in the present study, the mean distance between the ^125^I seed cavity and the nearest tumour deposit was 0.9 mm. While this does not exclude the possibility that tumour cells at closer distances were eradicated by the high local radiation dose, viable tumour cells were nonetheless observed near the ^125^I seed. Taken together, our findings suggest that any potential therapeutic effect of ^125^I seeds during NST is likely minimal and not clinically relevant.

In clinical practice, a variety of markers and placement timings are used in targeted axillary dissection procedures [12,[32], [33], [34]]. A systematic review by de Wild et al. identified six definitive markers: wire, ^125^I seed, 99mTc, (electro)magnetic/radiofrequency markers, black ink, and a clip [35]. Placement of markers can follow a one- or two-step procedure. In two-step procedures, the metastatic lymph node is marked pre-NST and re-localised after NST for definitive marker placement. De Wild et al. highlighted the draw-back of two-step procedures since the success rate to localise the pre-NST marked metastatic lymph node after NST on imaging varied from 48.8% to 100% [35]. Since our findings may indicate that leaving ^125^I seeds in place during NST has limited radioactivity effects in MARI-nodes, this could support a one-step localisation approach with ^125^I seeds.

This study has limitations. Molecular markers indicative of radiation-induced damage (such as caspase-3 or Bax/Bcl-2 [36]) were not evaluated. Therefore, more detailed insights into the radiobiological effects of radiation from ^125^I seeds used to mark lymph nodes on tumour cells and local tissue could not be assessed. Also, the evaluation of this study was restricted to lymph nodes containing residual disease, as potential therapeutic effects can only be assessed in the presence of residual disease, and did not include evaluation of the primary tumour or lymph nodes without residual disease. However, given the very low absorbed radiation dose observed in this study, it is unlikely that radiation from the ^125^I seeds contributes to achieving a complete response. While the findings of this study are probably generalisable to primary tumours and lymph nodes without residual disease, this remains to be confirmed. Moreover, current knowledge and criteria for histopathologic features that may reflect radiobiological effects of local radiation in lymph nodes marked with ^125^I seeds is limited, raising the possibility that important yet uncharacterised histopathologic features may have been overlooked. Furthermore, for the purpose of this study, a dose estimation with a simple point source model was used. Using a dedicated planning system for ^125^I seeds will likely give no deeper insight in the current research question. A similar approach was used in another study, although that study focused on foetal dose [37]. Notably, the mean ^125^I seed estimated absorbed radiation dose at 5 mm is 12.4 Gy but this dose should not be considered clinically equivalent to therapeutically used tumour bed boost doses as part of routine radiotherapy treatment. The area receiving the dose by the ^125^I seed is very small and limited to the immediate vicinity of the ^125^I seed, with rapidly decreasing doses at increasing distances. In addition, the dose by the ^125^I seed is delivered at low pace (half life time of 60 days), resulting in a very low biological effect due to DNA repair processes during dose deposition. Therefore, both the treated volume and radiobiological effect associated with ^125^I seeds differ substantially from those of therapeutically used tumour bed boost doses as part of routine radiotherapy treatment. Additionally, in the absence of established pathological criteria, the presence of giant cells inside a cavity within a lymph node became a defining criterion for a ^125^I seed cavity. However, this is not yet a validated standard in current pathology practice. Next, due to the practice of slicing lymph nodes into multiple sections, it was not always feasible to assess whether specific histopathologic features had originally been located close to the ^125^I seed or to perform precise distance measurements. Distance measurements were performed for residual disease when possible. However, tissue alterations such as fibrosis often lacked clear borders and were therefore not measured. Lastly, the significant associations for fibrosis and foamy histiocytes observed in the logistic regression analyses comparing MARI and non-MARI nodes were accompanied by wide 95% CIs, likely reflecting the limited sample size. Therefore, these findings should be interpreted with caution. Despite the present study's limitations, the study provides valuable preliminary insights that may inform and refine future research in this area.

To conclude, this study of patients with axillary residual disease following NST found differences in histopathologic features between MARI and non-MARI nodes as fibrosis, foamy histiocytes, and macrometastases were significantly more present in MARI nodes. In MARI nodes, ^125^I seed estimated absorbed radiation dose was not associated with the presence of fibrosis, foamy histiocytes, and macrometastases. Although ^125^I seeds appear to induce a mechanical reaction in MARI nodes, the therapeutic effect seems minimal and is not expected to be of clinical relevance.

## CRediT authorship contribution statement

**Florien J.G. van Amstel:** Writing – review & editing, Writing – original draft, Visualization, Validation, Resources, Methodology, Investigation, Formal analysis, Data curation, Conceptualization. **Janine M. Simons:** Writing – review & editing, Visualization, Resources, Methodology, Investigation, Conceptualization. **Loes Kooreman:** Writing – review & editing, Resources. **Melissa Lenaerts:** Writing – review & editing, Resources. **Sander M.J. van Kuijk:** Writing – review & editing, Resources, Methodology. **Lars H.P. Murrer:** Writing – review & editing, Resources, Methodology. **Cornelis M. de Mooij:** Writing – review & editing, Resources. **Ernest J.T. Luiten:** Writing – review & editing, Resources. **Carmen C. van der Pol:** Writing – review & editing, Resources. **Linetta B. Koppert:** Writing – review & editing, Resources. **Marjolein L. Smidt:** Writing – review & editing, Visualization, Resources, Methodology, Investigation, Conceptualization. **Paul J. van Diest:** Writing – review & editing, Visualization, Resources, Methodology, Investigation, Data curation, Conceptualization. **Thiemo J.A. van Nijnatten:** Writing – review & editing, Visualization, Supervision, Resources, Methodology, Investigation, Conceptualization.

## Data availability statement

Data and code for the final analyses are available from the corresponding author upon reasonable request.

## Ethical approval

The privacy and scientific committee of PALGA, the nationwide network and registry of histopathology and cytopathology in The Netherlands, provided approval for the current study (protocol number 2022-109).

## Sources of funding

Thiemo J.A. van Nijnatten and Florien J.G. van Amstel received funding for this study by the 10.13039/501100004622Dutch Cancer Society (REFINE-study; project 14055). None of the other authors received support from any organisation for the submitted work.

## Declaration of competing interest

The authors declare the following financial interests/personal relationships which may be considered as potential competing interests: Marjolein L. Smidt received institutional research funding not related to this study from Servier, Pharma, Nutricia and Illumina for the microbiota study. Thiemo J.A. van Nijnatten received speaker honoraria and institutional grant support and reports participation in medical advisory board meetings for Bayer and GE Healthcare, not related to the content of this study. Thiemo J.A. van Nijnatten reports participation in medical advisory board meetings for Screenpoint Medical, not related to the content of this study. Loes Kooreman received an educational fee not associated to the content of this study. If there are other authors, they declare that they have no known competing financial interests or personal relationships that could have appeared to influence the work reported in this paper.

## References

[1] Fisher B., Bryant J., Wolmark N., Mamounas E., Brown A., Fisher E.R. (1998). Effect of preoperative chemotherapy on the outcome of women with operable breast cancer. J Clin Oncol.

[2] Samiei S., Simons J.M., Engelen S.M., Beets-Tan R.G., Classe J.-M., Smidt M.L. (2021). Axillary pathologic complete response after neoadjuvant systemic therapy by breast cancer subtype in patients with initially clinically node-positive disease: a systematic review and meta-analysis. JAMA Surg.

[3] Simons J.M., van Nijnatten T.J.A., van der Pol C.C., van Diest P.J., Jager A., van Klaveren D. (2022). Diagnostic accuracy of radioactive iodine seed placement in the axilla with sentinel lymph node biopsy after neoadjuvant chemotherapy in node-positive breast cancer. JAMA Surg.

[4] Kuehn T., Bauerfeind I., Fehm T., Fleige B., Hausschild M., Helms G. (2013). Sentinel-lymph-node biopsy in patients with breast cancer before and after neoadjuvant chemotherapy (SENTINA): a prospective, multicentre cohort study. Lancet Oncol.

[5] Boileau J.-F., Poirier B., Basik M., Holloway C.M., Gaboury L., Sideris L. (2015). Sentinel node biopsy after neoadjuvant chemotherapy in biopsy-proven node-positive breast cancer: the SN FNAC study. J Clin Oncol.

[6] Boughey J.C., Suman V.J., Mittendorf E.A., Ahrendt G.M., Wilke L.G., Taback B. (2013). Sentinel lymph node surgery after neoadjuvant chemotherapy in patients with node-positive breast cancer: the ACOSOG Z1071 (alliance) clinical trial. JAMA.

[7] Caudle A.S., Yang W.T., Krishnamurthy S., Mittendorf E.A., Black D.M., Gilcrease M.Z. (2016). Improved axillary evaluation following neoadjuvant therapy for patients with node-positive breast cancer using selective evaluation of clipped nodes: implementation of targeted axillary dissection. J Clin Oncol.

[8] Straver M., Loo C., Alderliesten T., Rutgers E., Vrancken Peeters M. (2010). Marking the axilla with radioactive iodine seeds (MARI procedure) may reduce the need for axillary dissection after neoadjuvant chemotherapy for breast cancer. Journal of British Surgery.

[9] Zaorsky N.G., Davis B.J., Nguyen P.L., Showalter T.N., Hoskin P.J., Yoshioka Y. (2017). The evolution of brachytherapy for prostate cancer. Nat Rev Urol.

[10] Leite E.T.T., da Silva J.L.F., Capelletti E., Haddad C.M.K., Marta G.N. (2019). Prostate brachytherapy with iodine-125 seeds: analysis of a single institutional cohort. Int Braz J Urol.

[11] Janssen N., Nijkamp J., Alderliesten T., Loo C., Rutgers E., Sonke J. (2016). Radioactive seed localization in breast cancer treatment. Journal of British Surgery.

[12] Murthy V., Young J., Tokumaru Y., Quinn M., Edge S.B., Takabe K. (2021). Options to determine pathological response of axillary lymph node metastasis after neoadjuvant chemotherapy in advanced breast cancer. Cancers.

[13] Alamoodi M., Wazir U., Venkataraman J., Almukbel R., Mokbel K. (2024). Assessing the efficacy of radioactive iodine seed localisation in targeted axillary dissection for node-positive early breast cancer patients undergoing neoadjuvant systemic therapy: a comprehensive review and pooled analysis. Diagnostics.

[14] Han L., Li C., Wang J., He X., Zhang X., Yang J. (2016). Iodine-125 radioactive seed tissue implantation as a remedy treatment for recurrent cervical cancer. J Cancer Res Therapeut.

[15] Wang Z.M., Lu J., Zhang L.Y., Lin X.Z., Chen K.M., Chen Z.J. (2015). Biological effects of low-dose-rate irradiation of pancreatic carcinoma cells in vitro using 125I seeds. World J Gastroenterol.

[16] Ren F., Li B., Wang C., Wang Y., Cui B. (2022). Iodine-125 seed represses the growth and facilitates the apoptosis of colorectal cancer cells by suppressing the methylation of miR-615 promoter. BMC Cancer.

[17] Ju Z., Wang Z., Wang L., Li J., Wu Z., Li X. (2020). Experimental study on radiation damage of(125)I seeds implanted in canine gastric wall tissue. J Cancer Res Therapeut.

[18] Park C.K., Jung W.-H., Koo J.S. (2016). Pathologic evaluation of breast cancer after neoadjuvant therapy. Journal of pathology and translational medicine.

[19] Provenzano E., Bossuyt V., Viale G., Cameron D., Badve S., Denkert C. (2015). Standardization of pathologic evaluation and reporting of postneoadjuvant specimens in clinical trials of breast cancer: recommendations from an international working group. Mod Pathol.

[20] Newman L.A., Pernick N.L., Adsay V., Carolin K.A., Philip P.A., Sipierski S. (2003). Histopathologic evidence of tumor regression in the axillary lymph nodes of patients treated with preoperative chemotherapy correlates with breast cancer outcome. Ann Surg Oncol.

[21] Straub J.M., New J., Hamilton C.D., Lominska C., Shnayder Y., Thomas S.M. (2015). Radiation-induced fibrosis: mechanisms and implications for therapy. J Cancer Res Clin Oncol.

[22] Kim J.H., Jenrow K.A., Brown S.L. (2014). Mechanisms of radiation-induced normal tissue toxicity and implications for future clinical trials. Radiat Oncol J.

[23] van Nijnatten T.J., Simons J.M., Smidt M.L., van der Pol C.C., van Diest P.J., Jager A. (2017). A novel less-invasive approach for axillary staging after neoadjuvant chemotherapy in patients with axillary node-positive breast cancer by combining radioactive iodine seed localization in the axilla with the sentinel node procedure (RISAS): a Dutch prospective multicenter validation study. Clin Breast Cancer.

[24] Casparie M., Tiebosch A., Burger G., Blauwgeers H., Van de Pol A., Van Krieken J. (2007). Pathology databanking and biobanking in the Netherlands, a central role for PALGA, the nationwide histopathology and cytopathology data network and archive. Anal Cell Pathol.

[25] Bossuyt V., Provenzano E., Symmans W., Boughey J., Coles C., Curigliano G. (2015). Recommendations for standardized pathological characterization of residual disease for neoadjuvant clinical trials of breast cancer by the BIG-NABCG collaboration. Ann Oncol.

[26] Clark B.Z., Johnson R.R., Berg W.A., McAuliffe P., Bhargava R. (2023). Response in axillary lymph nodes to neoadjuvant chemotherapy for breast cancers: correlation with breast response, pathologic features, and accuracy of radioactive seed localization. Breast Cancer Res Treat.

[27] Donker M., Straver M.E., Wesseling J., Loo C.E., Schot M., Drukker C.A. (2015). Marking axillary lymph nodes with radioactive iodine seeds for axillary staging after neoadjuvant systemic treatment in breast cancer patients: the MARI procedure. Ann Surg.

[28] Nath R., Bice W.S., Butler W.M., Chen Z., Meigooni A.S., Narayana V. (2009). AAPM recommendations on dose prescription and reporting methods for permanent interstitial brachytherapy for prostate cancer: report of task group 137. Med Phys.

[29] Yorozu A. (2020). Current status of prostate brachytherapy in Japan. Jpn J Radiol.

[30] Sakurai T., Takamatsu S., Shibata S., Taka M., Ishiyama M., Yamazaki M. (2021). Incidence and dosimetric predictive factors of late rectal toxicity after low-dose-rate brachytherapy combined with volumetric modulated arc therapy in high-risk prostate cancer at a single institution: retrospective study. Brachytherapy.

[31] Sheetz M., Steiner C. (2018). Compliance with the US nuclear regulatory commission revised licensing guidance for radioactive seed localization. Health Phys.

[32] Reitsamer R., Peintinger F., Forsthuber E., Sir A. (2021). The applicability of magseed® for targeted axillary dissection in breast cancer patients treated with neoadjuvant chemotherapy. Breast.

[33] Natsiopoulos I., Intzes S., Liappis T., Zarampoukas K., Zarampoukas T., Zacharopoulou V. (2019). Axillary lymph node tattooing and targeted axillary dissection in breast cancer patients who presented as cN+ before neoadjuvant chemotherapy and became cN0 after treatment. Clin Breast Cancer.

[34] Balasubramanian R., Morgan C., Shaari E., Kovacs T., Pinder S.E., Hamed H. (2020). Wire guided localisation for targeted axillary node dissection is accurate in axillary staging in node positive breast cancer following neoadjuvant chemotherapy. Eur J Surg Oncol.

[35] de Wild S.R., Koppert L.B., van Nijnatten T.J., Kooreman L.F., Vrancken Peeters M.-J.T., Smidt M.L. (2024). Systematic review of targeted axillary dissection in node-positive breast cancer treated with neoadjuvant systemic therapy: variation in type of marker and timing of placement. Br J Surg.

[36] Jiao Y., Cao F., Liu H. (2022). Radiation-induced cell death and its mechanisms. Health Phys.

[37] Heeling E., van de Kamer J.B., Methorst M., Bruining A., van de Meent M., Vrancken Peeters M.-J.T. (2023). The safe use of 125I-seeds as a localization technique in breast cancer during pregnancy. Cancers.

