# Supplementary material for: Histopathological evaluation of local effects of radioactive iodine seeds in axillary lymph nodes in clinically node-positive breast cancer treated with neoadjuvant systemic therapy
Source: Breast. 2026 Mar 4;87:104746. doi: 10.1016/j.breast.2026.104746 (PMC13000488; doi:10.1016/j.breast.2026.104746)
Supplement: Multimedia component 1 [file mmc1.docx]

# **Supplementary**

**Table S1.** Formula of estimated absorbed radiation dose from a ^125^I seed

| **Dose** ($\mu Gy)$**=** $\boldsymbol{A}_{\boldsymbol{0}}\frac{\boldsymbol{\Gamma}}{\boldsymbol{d}^{\boldsymbol{2}}} \frac{\boldsymbol{t}_{\boldsymbol{1/2}}}{\boldsymbol{ln}\left( \boldsymbol{2} \right)} \left[ \boldsymbol{1-}\boldsymbol{e}^{\boldsymbol{-}\frac{\boldsymbol{t}}{\boldsymbol{t}_{\boldsymbol{1/2}}}\boldsymbol{ln(2)}} \right] \boldsymbol{e}^{\boldsymbol{-}\frac{\boldsymbol{d}}{\boldsymbol{d}_{\boldsymbol{1/2}}}\boldsymbol{ln(2)}}$ | |
| --- | --- |
| **A_0_** | = activity of the ^125^I seed at the day of placement (MBq) |
| **d** | = distance in tissue between the ^125^I seed and the measurement point (set at 5 mm) |
| ***d_1/2_*** | = half value thickness of ^125^I seeds (1.7 cm) |
| **t** | = time of ^125^I seed placement (days) |
| ***t_1/2_*** | = half life time of ^125^I seeds (60 days) |
| $\boldsymbol{\Gamma}$ | = source constant for ^125^I seed (0.034 $\left[ \mu Gy m^{2} \right]/\left[ MBq h \right]$) |

**
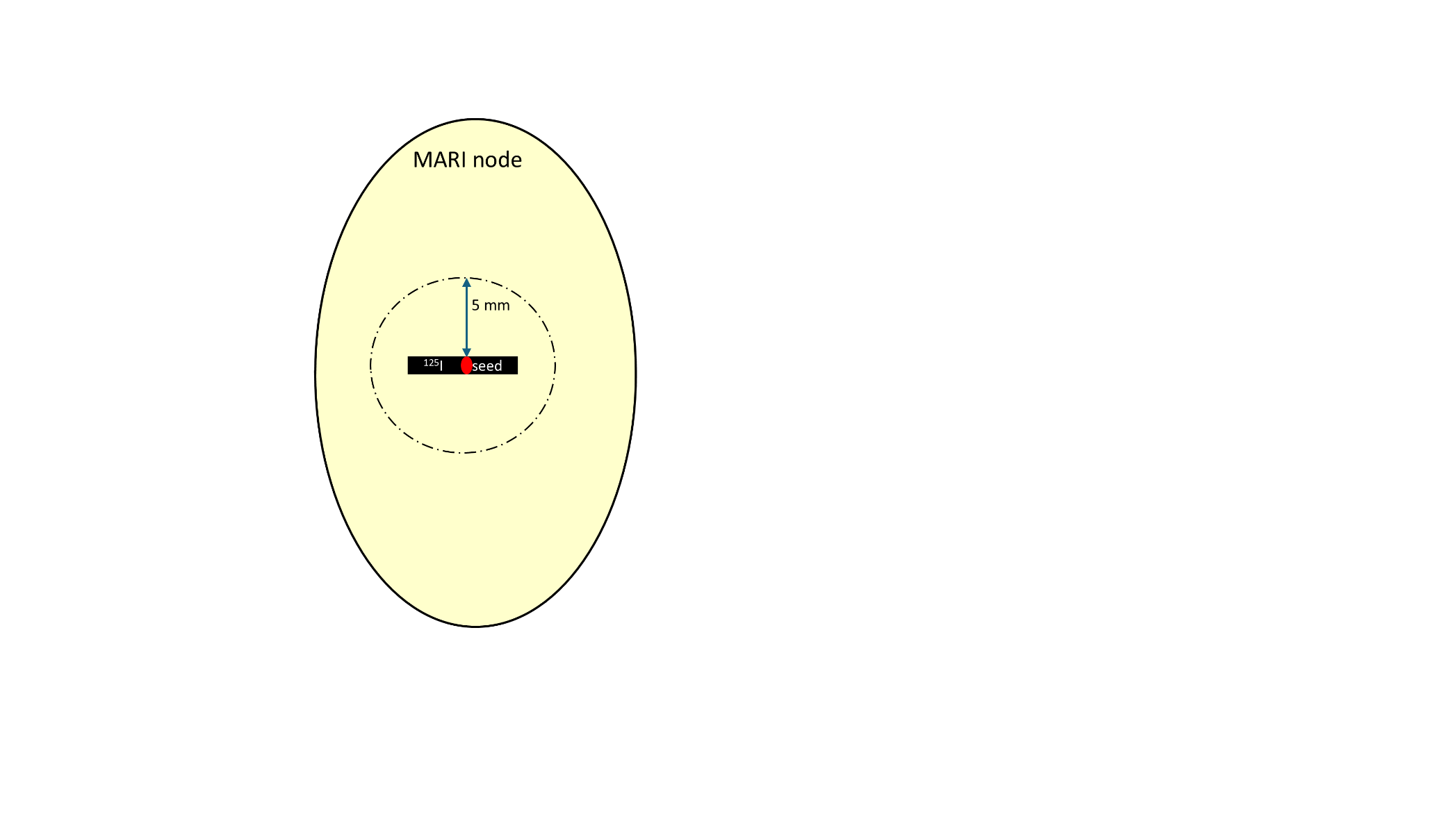
**

**Figure S1.** Schematic representation of a MARI node with a ^125^I seed. The dashed circle indicates the measurement point which was set at 5 mm. The red dot represents point source approximation of the ^125^I seed.
